# Conflicts of Interest during Contact Investigations: A Game-Theoretic Analysis

**DOI:** 10.1155/2014/952381

**Published:** 2014-04-14

**Authors:** Nicolas Sippl-Swezey, Wayne T. Enanoria, Travis C. Porco

**Affiliations:** ^1^Francis I. Proctor Foundation for Research in Ophthalmology, University of California, San Francisco, Box 0412, San Francisco, CA 94143-0412, USA; ^2^Department of Epidemiology and Biostatistics, University of California, San Francisco, Box 0412, San Francisco, CA 94143-0412, USA; ^3^Department of Ophthalmology, University of California, San Francisco, Box 0412, San Francisco, CA 94143-0412, USA

## Abstract

The goal of contact tracing is to reduce the likelihood of transmission, particularly to individuals who are at greatest risk for developing complications of infection, as well as identifying individuals who are in need of medical treatment of other interventions. In this paper, we develop a simple mathematical model of contact investigations among a small group of individuals and apply game theory to explore conflicts of interest that may arise in the context of perceived costs of disclosure. Using analytic Kolmogorov equations, we determine whether or not it is possible for individual incentives to drive noncooperation, even though cooperation would yield a better group outcome. We found that if all individuals have a cost of disclosure, then the optimal individual decision is to simply not disclose each other. With further analysis of (1) completely offsetting the costs of disclosure and (2) partially offsetting the costs of disclosure, we found that all individuals disclose all contacts, resulting in a smaller basic reproductive number and an alignment of individual and group optimality. More data are needed to understand decision making during outbreak investigations and what the real and perceived costs are.

## 1. Introduction

Contact investigation (contact tracing) is the identification of individuals who have come into contact with an infectious case and may be infected. The goals of contact tracing arise to reduce the likelihood of transmission (particularly to those individuals who are at greatest risk for developing complications of infection) and to identify individuals who are in need of medical treatment or other interventions [1]. Contact tracing has been used in the control of many diseases, including tuberculosis [2], smallpox [3], sexually transmitted diseases [4–6], influenza A (H7N2) [7], and severe acute respiratory syndrome (SARS) [8–12]. With the recent emergence of avian influenza A (H7N9) virus in humans in China [13, 14] and a novel coronavirus in the United Kingdom in connection with travel to the Middle East [15], contact tracing continues to play an important role in epidemiological investigations of emerging infectious diseases. As a result, contact tracing is a core component of epidemiological investigations, one of fifteen public health emergency preparedness and response capabilities of health departments (Capability 13: Public Health Surveillance and Epidemiological Investigation) [16].

Mathematical models have been used previously to evaluate the impact of contact investigations on the spread of infectious disease generally [17–25]. Others have focused on specific diseases including SARS [12], tuberculosis [26, 27], influenza [28], measles [29, 30], HIV [31, 32], gonorrhea [17, 33–36], chlamydia [36], and smallpox [21, 37–41]. The effectiveness, however, of contact tracing depends on the completeness of cooperation with contact elicitation. A previous qualitative study showed that miscommunication, misconceptions, and lack of trust in contact investigation staff can hinder the success of contact disclosure despite an individual's willingness to identify contacts [42]. In addition, individuals involved in illegal and/or illicit social connections, including drug use [43], gambling [44], and extramarital affairs [45], may fear loss of anonymity and may, for this reason, fail to cooperate in naming contacts.

Individuals face costs—real or perceived—of contact disclosure. Real costs include time spent in interviews and in the effort spent recalling contacts. While contact investigations are and must be conducted in a manner that protects confidentiality, interviewees may perceive disclosure as a privacy risk, which may create a perceived cost. While the perceived and real costs of disclosure and their impacts on early contact investigation have been documented, the effects have not been explored thoroughly. If the disclosure of contacts provides a public benefit for disease control, but individuals perceive a cost for disclosing contacts, then there may be a conflict between real or perceived individual interests and the public good.

Mathematical models of contact tracing and ring vaccination—which requires contact tracing—have explored the effect of success rates of contact tracing that are less than unity and thus incorporate less than complete cooperation with contact elicitation [12, 20, 21, 40]. They have not, however, explored strategic nondisclosure of contacts or the role of perceived costs in that strategic behavior. Game theory has been used in other investigations of disease transmission [46–52], especially for vaccines [49, 53–58], treatment decisions [59], and the use of social distancing during an epidemic [60]. The impact of strategic behavior has been explored in the context of ring vaccination, which requires contact investigation [61–63]. These analyses, however, examine vaccination choice and do not explore disclosure choice. In this paper, we develop a simple mathematical model of contact investigations among a small group of individuals and apply game theory to explore conflicts of interest that may arise in the context of perceived costs of disclosure. We determine whether or not it is possible for individual incentives to fa-vor noncooperation, even though cooperation would yield a better group outcome.

## 2. Methods

### 2.1. Overview

Our analysis is based on a stochastic, continuous-time process taking place in a small social group; we formulate the model in general terms and restrict our analysis to a group of size 3. Such a model of a small group may, for instance, describe small groups within a model featuring more transmission between members of the same group, but allowing between-group transmission for any two individuals in the population [64, 65]. We will use this model to derive the expected reduction in infection risk for an individual and expected costs.

### 2.2. Transmission Model in the Absence of Contact Investigation

We assume a standard SEIR model for the untreated natural history of the disease [66]. The specific disease is left unspecified for this paper. In particular, we assume that infected individuals are latently infected for a period prior to the onset of symptoms and, for simplicity, that only symptomatic individuals are infectious. Infectious individuals are then assumed to recover with full immunity. Susceptible individuals become newly infected at a rate which depends on the number of infected individuals with whom they come into contact.

Infectious individuals are always assumed to be diagnosed and isolated or treated, and we assume that such individuals are no longer causing new infections in the population. Such individuals may be undergoing treatment which reduces or eliminates infectivity or may be isolating themselves from others during the time of infectiousness. In the absence of contact investigation, the process may be described by the following states: *S* (susceptible), *E* (exposed or latent), *I* (untreated, infectious), and *R* (removed). Exposed individuals in state *E* progress to infectiousness *I* at rate *γ* and infectious cases *I* are diagnosed and removed at rate *ρ* due to symptoms. Individuals in the small group experience a force of infection from both within and from outside the group, though we will ignore infection from outside the group. The force of infection from within the group will be given by *λ*(*t*) = *βY*(*t*)/(*N* − 1), where *Y* is the number of infectives in the group. Our analysis concerns transmission events following the introduction of a single case in a small group (of size 3). Similar models have been analyzed by many other authors.

The following equations describe a single group in the absence of contact investigation. Let *X*_*i*_ be the state of individual *i* in the small group (here, *X*_*i*_ must be either *S*, *E*, *I*, or *R*). Then let *q*_*X*_1_,*X*_2_,*X*_3_,…,*X*_*N*__ be the probability that individual 1 is in state *X*_1_, individual 2 is in state *X*_2_, and so on; for example, for the case *N* = 3, *q*_*I*,*S*,*S*_ is the probability that person 1 is infectious, and persons 2 and 3 are susceptible. We assume that the transition rates for all the individuals in the population are conditionally independent of each other given the current state, so that the total rate of departure from the current state is the sum of the rates with which each individual leaves the current state he or she is in.

For the case *N* = 3, in the absence of contact investigation, we have 4^3^ = 64 possible states for the small group. Since we are ignoring exogenous transmission, (*d*/*dt*)*q*_*S*,*S*,*S*_ = 0. Because each individual exposed person progresses with rate *γ*,

(1) $$\begin{array}{c} \frac{d}{dt}q_{E,S,S}=-\gamma q_{E,S,S} \end{array}$$

(with a similar equation for *q*_*S*,*E*,*S*_ and for *q*_*S*,*S*,*E*_).

We denote the transmission coefficient by *β*, so that we have

(2) $$\begin{array}{c} \frac{d}{dt}q_{I,S,S}=\gamma q_{E,S,S}-\rho q_{I,S,S}-2\frac{\beta }{2}q_{I,S,S}, \end{array}$$

since (1) we have assumed that the recovery time for the infective (an exponential with rate *ρ*) and the infection times for each susceptible are independent and that (2) the force of infection for each susceptible individualsis given by *β* times the prevalence in the rest of the population (the number of the infective divided by the population size minus 1).

The full set of equations for a single small group can be written in a more compact form. Let 1_*X*_*i*_=*S*_ be the indicator function for the event that person *i* is in state *S* and so forth. For the *N* = 3 case, we let *X*_1_, *X*_2_, and *X*_3_ represent the state of person 1, 2, and 3, respectively. For all states *X*_1_, *X*_2_, and *X*_3_,

(3) ddtqX1,X2,X3 =γ(1X1=I−1X1=E)qE,X2,X3  +γ(1X2=I−1X2=E)qX1,E,X3  +γ(1X3=I−1X3=E)qX1,X2,E  +ρ(1X1=R−1X1=I)qI,X2,X3  +ρ(1X2=R−1X2=I)qX1,I,X3  +ρ(1X3=R−1X3=I)qX1,X2,I  +λ(X1,X2,X3)(1X1=E−1X1=S)qS,X2,X3  +λ(X1,X2,X3)(1X2=E−1X2=S)qX1,S,X3  +λ(X1,X2,X3)(1X3=E−1X3=S)qX1,X2,S,

where *λ*(*X*_1_, *X*_2_, *X*_3_) = *β*∑_*i*=1_^3^1_*X*_*i*_=*I*_/(*N* − 1). Extension to larger sizes *N* for the small groups is straightforward.

### 2.3. Contact Investigation Model in the Absence of Infection

Before extending the simple model (3) to include contact investigation, we introduce the contact investigation process model that would occur in the absence of anyone being infected (as might occur, for instance, in an investigation of a suspected case subsequently determined not to be truly infected).

Here, all individuals are susceptible, but we assume that individuals are either unknown to the investigation (*S*) or known (*S*′). In the absence of infection, individuals become newly known when and only when they are disclosed by other known individuals. Once an individual is known to the investigation, he or she remains known throughout the investigation. In effect, we assume contact investigation behaves like simple SI epidemic itself.

For disease transmission, we assume that disease may be transmitted between any two people. For contact investigation, we do not assume that every person is willing to disclose any other person; any identified person will be asked to name all contacts but may choose not to do so. Let *δ*_*ij*_ be a binary variable indicating whether person *i* would disclose *j* if investigated. It is possible that *δ*_*ij*_ ≠ *δ*_*ji*_; for instance, person *i* is willing to disclose person *j*, but person *j* is not willing to disclose person *i*. We assume that the disclosure variables *δ*_*ij*_ are constant in time and do not depend on the state of the system; whether person *i* is willing to disclose person *j* does not depend on whether or not person *j* has disclosed person *i*, would be willing to disclose person *i*, or has already disclosed person *i*. While no individual is epidemiologically isolated, it is possible that there is an individual whom no one would disclose; such a person could only become known to investigators if he or she were diagnosed first.

We model the rate at which persons unknown to the investigation become newly known as follows. Suppose that person *i* is known to the investigation, but person *j* is not. Then if person *i* is willing to disclose person *j*, then we assume that the waiting time for person *j* to become known is exponentially distributed with rate *ξ* as a result, independent of whether person *i* is willing to disclose any other individuals. For mathematical simplicity, we assume that the rate at which any unknown individual becomes known is the sum of the rates corresponding to each contact who is disclosing him or her; we denote the total rate of investigation for person *i* by *η*_*i*_. No specific order is assumed for the investigations to take place.

In this setting of a small group of three people without any infection, *q*_*S*,*S*,*S*_ denotes the probability that no individual has been contacted by disease control investigators, that is, that no individual is known to the investigation. Beginning with individuals who are known to the investigation at the beginning (*t* = 0), new individuals become known when they are disclosed by people already known, and so if no one is assumed known at the beginning, no one will ever become known. Moreover, since we assume that once a person is known to the investigation, he or she remains known; (*d*/*dt*)*q*_*S*,*S*,*S*_ = 0. Continuing, *q*_*S*′,*S*,*S*_ is the probability that person 1 is known but that persons 2 and 3 are not known. Since the rate at which one person will become known as the result of being disclosed by a single other individual is *ξ*, the rate at which person 2 will be disclosed is *ξδ*_12_. We assume an independent and identical rate for the identification of person 3 as a result of person 1. We can then write

(4)ddtqS′,S,S =−qS′,S,S(ξδ12+ξδ13),(5)ddtqS′,S′,S =qS′,S,Sξδ12+qS,S′,Sξδ21−qS′,S′,Sξ(δ13+δ23),ddtqS′,S′,S′=ξ(qS′,S′,S(δ13+δ23)+qS′,S,S′(δ12+δ32)  +qS,S′,S′(δ21+δ31))

with similar equations for (*d*/*dt*)*q*_*S*,*S*′,*S*_, (*d*/*dt*)*q*_*S*,*S*,*S*′_, (*d*/*dt*)*q*_*S*′,*S*,*S*′_, and (*d*/*dt*)*q*_*S*,*S*′,*S*′_.

### 2.4. Disease Transmission and Contact Investigation

We will add the contact investigation model from the previous subsection to the simple SEIR transmission model. One way for individuals to become known to the investigation is to be disclosed by another known individual who is willing to disclose him or her, as in the previous subsection. We now assume, additionally, that reporting insures that all diagnosed individuals are known to the investigation, and we ignore reporting delays. Newly diagnosed individuals are the only way that a contact investigation can become initiated; the first diagnosed individual inaugurates the first contact investigation, regardless of whether any other individuals have been infected or diagnosed and regardless of whether or not the first diagnosed individual was the first infectious case. (We do not assume that any individuals are known to the investigation at the outset (*t* = 0).)

When an individual is investigated, several events occur in addition to begin queried about his or her contacts (who will then be investigated at rate *ξ* if disclosed), as in Section 2.3. First, if an individual is investigated and is infective (*I*), he or she is immediately diagnosed. Thus, the mean time to diagnosis can be shorter for an infective if she or he has contacts that disclose her or him.

When a susceptible individual is investigated, he or she may take protective measures to reduce the chance of infection. Also, when an exposed individual is contacted, he or she may receive postexposure protective measures. Such measures may include vaccination (as in the case of measles or smallpox) or the provision of immunoglobulin (as in the case of measles, for instance). Thus, susceptible individuals who are known to the investigation are assumed to have a smaller risk of infection, and both susceptible and exposed individuals known to the investigation have a rate of vaccination or other protective actions which may prevent them from becoming cases. For an individual in state *S*′, we assume that the efficacy of personal protective measures in reducing the risk of infection is denoted 1 − *ζ*, so that if *ζ* = 0, the person has no risk at all, and if *ζ* = 1, the protective measures are completely without effect. The force of infection experienced by a person in state *S*′ is then given by *ζλ*(*X*_1_, *X*_2_, *X*_3_). Individuals in states *S*′ receive postexposure prophylaxis or vaccination at rate *ω*, and can thus be protected from disease, entering state *V*.

Finally, we assume that any exposed person (state *E*′) contacted during an investigation is assumed to have been made aware that he or she may have been exposed. Such individuals are vaccinated at rate *ω*, just as susceptible individuals are, and, moreover, such individuals are assumed to be diagnosed and removed immediately if they develop symptoms (and are therefore never infectious to others). Thus, in our simple idealization of contact investigation, we assume that contact investigations help control disease by (1) preventing transmission from infections that occur in contacted individuals prior to symptoms due to rapid diagnosis and voluntary isolation, (2) permitting the use of postexposure protective measures for exposed persons, and (3) allowing uninfected susceptible individuals to take protective measures. An individual who is never infected and never disclosed will never become known to an investigation. Finally, we assume no further attrition; all named contacts will eventually be identified.

**Figure 1:**
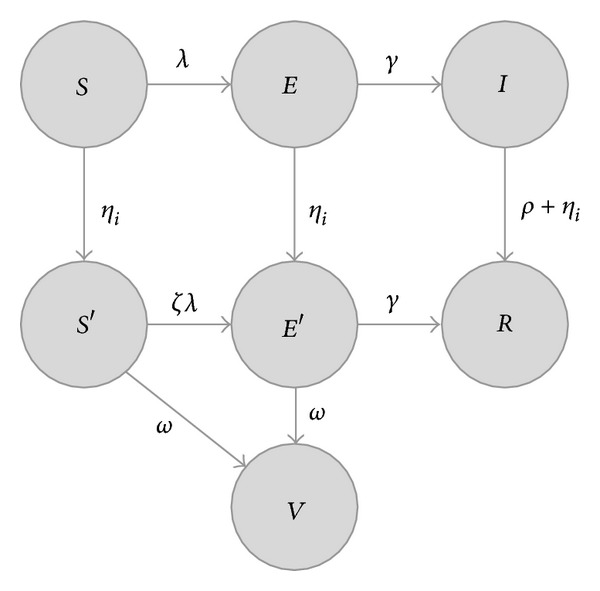
State space for a single individual, according to (6). Each possible state is represented with a circle, labeled with the state (*S*—susceptible, *E*—exposed, *I*—infectious, *R*—diagnosed and visited by a disease control investigator, *S*′—susceptible but has been visited by a disease control investigator, *E*′—exposed, but has been visited by a disease control investigator, *V*—exposed, but protected by postexposure prophylaxis). Possible transitions are indicated with arrows and informally labeled with expressions used to compute the rate; see (6) for details. We denote the total force of infection for each individual by *λ*, which depends on the number of other infected individuals; we denote the total rate of investigation for a given individual by *η*_*i*_, which depends on the number of other investigated individuals willing to disclose that individual.

The state space of the model now may be written (see Figure 1) as follows:

 
*S*—susceptible, never contacted by disease control investigators,
 
*E*—exposed, never contacted by disease control investigators,
 
*I*—infectious, never contacted by disease control investigators,
 
*S*′—susceptible that* has been* contacted by disease control investigators,
 
*E*′—exposed,* and has been* contacted by disease control investigators,
 
*R*—diagnosed and removed, and has been contacted by disease control investigators (by assumption),
 
*V*—exposed but removed; disease prevented due to post-exposure prophylaxis or vaccination.

Specifically, an individual in state *S* (susceptible, never investigated) may move to state *S*′ (susceptible, investigated); the rate at which this occurs depends on which contacts have been investigated and whether the contacts choose to disclose. Suppose that person 1 is in state *S* (and thus has not been investigated). If person 2 is in state *S*′, *E*′, *R*, or *V*, then person 2 has been visited by disease control investigators and has had an opportunity to disclose person 1 (as well as person 3) to the investigators. Similarly, if person 3 is in one of *S*′, *E*′, *R*, or *V*, he or she too has an opportunity to disclose person 1 (as well as person 2). The total rate at which person 1 will be visited is then *η*_1_ = *δ*_21_1_*X*_2_∈{*S*′,*E*′,*R*,*V*}_ + *δ*_31_1_*X*_3_∈{*S*′,*E*′,*R*,*V*}_. When a person in state *S* is visited, he or she moves to the state *S*′; when a person in state *E* is visited, he or she moves to *E*′, and when an infective, in state *I*, is visited, he or she is diagnosed and enters state *R*.

For the case *N* = 3, we may write the equations in the same compact form as above. The equation below (representing all 7^3^ = 343 states of the process) includes terms featuring *γ* for individuals progressing from latency, terms featuring *ρ* for removal of the infective, terms featuring *λ*(*X*_1_, *X*_2_, *X*_3_) for disease transmission within the cluster, terms featuring *ω* for postexposure preventive measures, and terms featuring *δ*_*ij*_ for disclosure of contacts.

We write for all states *X*_1_, *X*_2_, and *X*_3_ (where *X*_*i*_ ∈ {*S*, *E*, *I*, *S*′, *E*′, *R*, *V*}),

(6) $$\begin{array}{c} \frac{d}{dt}q_{X_{1},X_{2},X_{3}}=q_{B}^{\prime }+q_{A}^{\prime }+q_{R}^{\prime }+q_{I}^{\prime }+q_{V}^{\prime }+q_{C,1}^{\prime }+q_{C,2}^{\prime }+q_{C,3}^{\prime }, \end{array}$$

where *q*_*B*_′ are terms for disease progression before a person is ever contacted in an investigation, *q*_*A*_′ are terms for disease progression after a person has been contacted, *q*_*R*_′ are terms for removal by diagnosis unrelated to contact investigation, *q*_*I*_′ are terms for infection, *q*_*V*_′ are terms for vaccination, and *q*_*C*,*i*_′ (*i* = 1,2, 3) are terms for disclosure and contact investigation. This, like the previous set, may be straightforwardly extended to larger group sizes.

Here, for *N* = 3,

(7) qB′=γ(1X1=I−1X1=E)qE,X2,X3+γ(1X2=I−1X2=E)qX1,E,X3 +γ(1X3=I−1X3=E)qX1,X2,E,qA′=γ(1X1=R−1X1=E′)qE′,X2,X3 +γ(1X2=R−1X2=E′)qX1,E′,X3 +γ(1X3=R−1X3=E′)qX1,X2,E′,qR′=ρ(1X1=R−1X1=I)qI,X2,X3+ρ(1X2=R−1X2=I)qX1,I,X3 +ρ(1X3=R−1X3=I)qX1,X2,I.

Individuals in *S* and *S*′ can both be infected, so that the infection component has six terms:

(8) qI′=λ(X1,X2,X3)(1X1=E−1X1=S)qS,X2,X3 +λ(X1,X2,X3)(1X2=E−1X2=S)qX1,S,X3 +λ(X1,X2,X3)(1X3=E−1X3=S)qX1,X2,S +ζλ(X1,X2,X3)(1X1=E′−1X1=S′)qS′,X2,X3 +ζλ(X1,X2,X3)(1X2=E′−1X2=S′)qX1,S′,X3 +ζλ(X1,X2,X3)(1X3=E′−1X3=S′)qX1,X2,S′,

*λ*(*X*_1_, *X*_2_, *X*_3_) = *β*∑_*i*=1_^3^1_*X*_*i*_=*I*_/(*N* − 1).

Individuals in both *S*′ and *E*′ can be protected by vaccination:

(9) qV′=ω(1X1=V−1X1=S′)qS′,X2,X3 +ω(1X2=V−1X2=S′)qX1,S′,X3 +ω(1X3=V−1X3=S′)qX1,X2,S′ +ω(1X1=V−1X1=E′)qE′,X2,X3 +ω(1X2=V−1X2=E′)qX1,E′,X3 +ω(1X3=V−1X3=E′)qX1,X2,E′.

For investigation, we assume that person 1 becomes investigated at rate *ξ* if person 2 is a known case or contact (is in state *S*′, *E*′, *R*, or *V*) and is willing to disclose person 1 (*δ*_21_ = 1) and at an additional rate *ξ* if person 3 is a known case or contact willing to disclose him or her. Thus,

(10) qC,1′=ξ(δ211X2∈{S′,E′,R,V}+δ311X3∈{S′,E′,R,V}) ×((1X1=S′−1X1=S)qS,X2,X3   +(1X1=E′−1X1=E)qE,X2,X3   +(1X1=R−1X1=I)qI,X2,X3)

Similarly for person 2,

(11) qC,2′=ξ(δ121X1∈{S′,E′,R,V}+δ321X3∈{S′,E′,R,V}) ×((1X2=S′−1X2=S)qX1,S,X3   +(1X2=E′−1X2=E)qX1,E,X3   +(1X2=R−1X2=I)qX1,I,X3)

and person 3,

(12) qC,3′=ξ(δ131X1∈{S′,E′,R,V}+δ231X2∈{S′,E′,R,V}) ×((1X3=S′−1X3=S)qX1,X2,S    +(1X3=E′−1X3=E)qX1,X2,E    +(1X3=R−1X3=I)qX1,X2,I).

**Figure 2:**
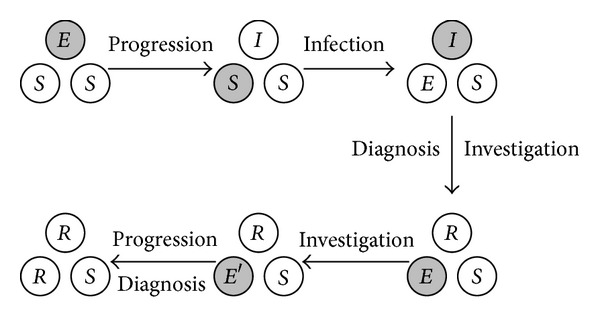
Example of state transitions within a small group, according to (6). Each arrow is labeled with a description of the transition. Each individual is represented as a circle, labeled with the state (*S*—susceptible, *E*—exposed, *I*—infectious, *R*—diagnosed and visited by a disease control investigator, *S*′—susceptible but has been visited by a disease control investigator, *E*′—exposed, but has been visited by a disease control investigator, *V*—exposed, but protected by post-exposure prophylaxis). The individual who undergoes the next transition is shown as a gray circle. Many such paths are possible.

Equation (6) describes a continuous time Markov process [67] for stochastic transitions among the 7^3^ possible states of a three-person group. The equations imply that the transitions between the states form a directed acyclic graph; no state can ever be visited more than once. Thus, beginning with a single index case (person 1 without loss of generality), the system undergoes stochastic transitions until it reaches an absorbing state.  Figure 2 provides an example of one such trajectory. First in this example, person 1 is exposed and then becomes infectious. In the second step, person 1 infects person 2 (lower left circle). Person 1 is diagnosed and a contact investigation occurs in the third step. Person 2 is then contacted and investigated in the fourth step but then progresses to disease and diagnosis in the last step. In this example, person 3 never becomes infected.

At time 0, *q*_*E*,*S*,*S*_(0) = 1, and all other states, *q*_*X*_1_,*X*_2_,*X*_3__(0) = 0 (for all *X*_1_, *X*_2_, and *X*_3_ such that (*X*_1_, *X*_2_, *X*_3_)≠(*E*, *S*, *S*)). The final probabilities can be computed by integrating this set of first order linear equations with constant coefficients (6) to determine the solution for *t* → *∞*. The expected complete size of a within-group outbreak may be found by

(13) μ=qR,X2∈{S,S′},X3∈{S,S′}(∞) +2(qR,X2∈{S,S′},R(∞)+qR,R,X3∈{S,S′}(∞)) +3qR,R,R(∞),

where *q*_*R*,*X*_2_∈{*S*,*S*′},*X*_3_∈{*S*,*S*′}_(*t*) = *q*_*R*,*S*,*S*_(*t*) + *q*_*R*,*S*,*S*′_(*t*) + *q*_*R*,*S*′,*S*_(*t*) + *q*_*R*,*S*′,*S*′_(*t*) for any *t* and so forth. Although the number of individuals in each state is always an integer, the expected values we compute are not. For the *N* = 3 case, the above equations imply that in the absence of disclosure (*δ*_*ij*_ = 0 for all *i*, *j*),

(14) $$\begin{array}{c} \mu =\frac{\rho }{\beta +\rho }+2\frac{4\beta \rho^{2}}{\left(\beta +\rho \right){\left(\beta +2\rho \right)}^{2}}+3\frac{\left(\beta +4\rho \right)\beta^{2}}{\left(\beta +\rho \right){\left(\beta +2\rho \right)}^{2}}. \end{array}$$

The nature of the costs or disutilities associated with either disclosure or disease is not specified. Disclosure in some settings is an undesirable outcome, and we wish to compare this to the costs of disease. It is not necessary that a person actually incurs any harm from the investigation, because, for some individuals, even a confidential disclosure of an illicit contact may be uncomfortable and undesirable. In principle, it may be possible to estimate such costs using willingness to pay data or time-tradeoff data, but we do not pursue this here.

We assume that the cost of disclosure is *C*_*ij*_, which is the cost incurred by person *i* upon disclosing person *j*. We assume that this cost is incurred whenever person *i* is investigated and has chosen to disclose person *j*, regardless of how the person is actually found (whether or not person *j* is diagnosed before being reached by an investigation, or whether or not he or she has been disclosed by someone else). We will assume an overall cost of participating in any disease control investigation (and this cost may be zero or even negative, in case of an incentive for participation); denote this overall cost by *C*_*o*_; we will assume that this is zero in almost all cases below unless specifically indicated otherwise. We denote the cost of infection by *F*, and always assume *F* > *C*_*o*_ (disease is always costlier than any incentive for participating in a contact investigation).

Our assumptions imply that the payoff for each person may be computed from the final state of the system. For any final state represented by (*X*_1_, *X*_2_, *X*_3_), where *X*_*i*_ is the state of person *i*, the payoff for person *i* given that state may be computed from the negative of the cost:

(15) $$\begin{array}{c} P_{i}^{\left(X_{1},X_{2},X_{3}\right)}=-\left(1_{X_{i}=R}F+1_{X_{i}\in \{S^{\prime },R,V\}}\left(\overset{N}{\underset{j=1}{\sum }}\delta_{ij}C_{ij}+C_{o}\right)\right), \end{array}$$

where *δ*_*ii*_ = 0. Here, if a person is investigated, we compute the disclosure costs for each person she or he has chosen to disclose. If a person was infected, the final state is *R*, and we add the cost for infection *F*. Finally, we add the overall participation cost *C*_*o*_. The net expected payoff for person *i* is then obtained by summing the payoffs for each final state *P*_*i*_^(*X*_1_,*X*_2_,*X*_3_)^ over all possible final states:

(16) Pi=∑X1∈{S,S′,R,V}∑ X2∈{S,S′,R,V}∑ X3∈{S,S′,R,V}qX1,X2,X3(∞)Pi(X1,X2,X3).

The payoff for the entire group is simply *P* = ∑_*i*_ *P*_*i*_.

Alternatively, we may assume that the cost for each person is

(17) Pi(X1,X2,X3) =−(1Xi=RF+1Xi∈{S′,R,V}(∑j=1NδijCij+Comax⁡(δij))),

where, in this case, the overall cost or benefit *C*_*o*_ is assumed to occur only if the respondent *i* actually discloses a contact. Other cost models are possible; for instance, it is possible that an individual could incur a cost if someone else discloses him or her. For this paper, we consider only the simple model outlined here.

If we assume that each individual infects *K* individuals outside their group and that the population is composed of many such groups, then the overall basic reproduction number, describing the ability of a disease to invade the population as a whole, is given by *Kμ*, as shown by [64]. Contact investigation acts to control the disease, in this simple setting, by reducing *μ*. In general, contact investigations may overlap groups, which are not included in the simple model above. In the analysis that follows, we distinguish the payoff for the individuals separately and for the small group (*N* = 3) as a whole; we do not treat society at large (persons outside the group we are modeling).

## 3. Results

The system of ordinary differential equations given by (6) is a linear system with constant coefficients. Beginning with the initial condition *q*_*X*_1_,*X*_2_,*X*_3__ = 0 for all combinations of *X*_1_, *X*_2_, and *X*_3_ other than *q*_*E*,*S*,*S*_ = 1, the total probability in each final state of the system as *t* → *∞* can be computed as the sum of the probability of arriving at each final (i.e., absorbing) state along each possible path to that state. For simplicity of discussion, we use the conventional names Alice, Bob, and Charlie for persons 1, 2, and 3, respectively; these widely used conventional names have no other significance (e.g. [68]). We computed the total infection probability for each person, assuming that the epidemic begins with Alice exposed. Not all of the total 343 system states are ultimately reachable from the initial condition (assuming *δ*_*ij*_ > 0 for *i*, *j* = 1,2, 3 and *i* ≠ *j*). We will ignore boundary cases corresponding to no infection, recovery, investigation, and progression; we always assume *β* > 0,  *γ* > 0,  *ξ* > 0, and *ρ* > 0.

Equation (6) defines a continuous time Markov chain. For all possible values of the decision variables *δ*_*ij*_, the chain always exhibits absorbing states. Specifically, a triple (*X*_1_, *X*_2_, *X*_3_) specifying the states of each individual can only be an absorbing state if *X*_*i*_ ∈ {*S*, *S*′, *R*, *V*} for *i* = 1,2, 3, because there is always a nonzero transition rate from any state containing an individual in states *E*, *E*′, or *I* (Figure 1). Any triple (*X*_1_, *X*_2_, *X*_3_) where *X*_*i*_ ∈ {*S*, *S*′, *R*, *V*} (*i* = 1,2, 3) can represent an absorbing state for the entire system if for all *k* such that *X*_*k*_ = *S*_*k*_, *δ*_*jk*_ = 1 implies *X*_*j*_ = *S*_*j*_, which simply states that an absorbing state for the system containing an uninvestigated susceptible individual is only possible if the only people willing to disclose him or her are themselves uninvestigated susceptible individuals. States containing *S*′ are absorbing states only when *ω* = 0.

The transition rates from each state of the system to each other state of the entire system constitute the generator *P* of the system. We let *p*_*lk*_ be the transition rate to state *l* from state *k*; *p*_*kk*_ = 0 for all *k*. We then define the usual jump chain [69] associated with the continuous time Markov chain defined by *P*, that is, a discrete time Markov chain which corresponds to the sequence of state transitions. The set of states may be divided into transient states and absorbing states, and we will arrange the states such that (1) the initial state (*E*, *S*, *S*) is first and (2) if *p*_*lk*_ > 0, state *k* comes before state *l*. The absorbing states therefore come last. The probability matrix for the jump chain can then be written in partitioned form

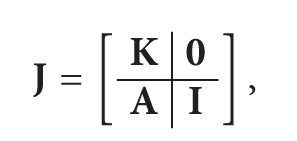
(18)
where the leading block **K** corresponds to all the transient states of the system and **A** to transitions from the transient states to the absorbing set. The probability that the system enters an absorbing state *l* given being in any transient state initially is then **A**(**I** − **K**)^−1^. Since (**I** − **K**)^−1^ = **I** + **K** + **K** ^2^ + ⋯, the expression (**I** − **K**)^−1^ can be interpreted as a sum over all possible paths from the initial state to the penultimate transient state; **A**(**I** − **K**)^−1^ is then the sum over all paths from the initial state to the absorbing state. It can be shown that **K** is acyclic.

In practice, we expressed the elements of the jump matrix **J** as symbolic expressions, represented by clauses in a Prolog knowledge base (http://www.swi-prolog.org/, v. 6.2.6 for Macintosh). This was then used to enumerate all possible sequences of system states, together with the conditional probabilities that the system would undergo a transition to the next state in the sequence given the current state. This computation was conducted for each of the 64 strategy choices of the three players (not each strategy choice corresponds to the same list of absorbing states for the model; when there is no disclosure, for instance, states such as (*I*, *S*, *V*) cannot be reached). Finally, algebraic simplification of the resulting path probabilities was performed using the computer mathematics packages Sage (http://www.sagemath.org/, v. 5.0 for Macintosh) and Form (http://www.nikhef.nl/~form/maindir/, v. 4.0). These were checked using numerical integration using the package   deSolve for R (http://www.r-project.org/, v. 3.0.1 for Macintosh).

For each strategy choice of all three individuals, we determined the probability that person 3 (Charlie) was infected. Assuming fixed strategies for the other two individuals, how does the infection probability for Charlie change if he chooses to disclose other individuals? The results are summarized in Table 1. A similar table can be written for the second individual (Bob, not shown).

Equations (19)–(22) provide analytic expressions for the change in infection probability experienced by person 3 (Charlie) for each combination of disclosure choices made (as given in Table 1). Using these infection probabilities, we can compute the expected benefits as well as costs experienced by each person. These costs and benefits depend on the choices made by each person in the group and can be used to compute the payoff for each player and therefore the solution to the game

(19) K1=4β2γ2ρξ2ζ((γ+ω)(β+ρ)(β+2ρ)×(β+2ρ+2ξ)(β+2γ+2ρ)×(βζ+2ω+2ρ)(βζ+2ω+2ρ+2ξ))−1K2=β2γ2ρξ2ζ(c0+c1β+c2β2+c3β3)  ×((γ+ξ)(γ+ω)2(β+ρ)(β+2ρ)(γ+ω+ξ)×(β+2ρ+2ξ)(β+2γ+2ρ)(βζ+2ω+2ρ)×(βζ+2ω+2ρ+2ξ))−1.

Here,

(20) c0=4(γ3+2γ2ω+2γ2ρ+2γ2ξ+γω2+4γωρ+3γωξ+2γρ2+4γρξ+γξ2+2ω2ρ+ω2ξ+4ωρ2+6ωρξ+ωξ2+2ρ3+4ρ2ξ+2ρξ2),c1=4(ρ2ζ+ρξζ+γ(γ+2ω+2ρ+2ξ)+ω(ω+4ρ+3ξ)+2ρ2+3ρξ+ξ2), c2=2(2ρζ+ξζ+γ+2ω+ρ+ξ),

and *c*_3_ = *ζ*. Also,

(21) K3=4β2γ2ρξ2ζ((γ+ω)(β+ρ)(β+2ρ)(β+2ρ+4ξ)×(β+2γ+2ρ)(βζ+2ω+2ρ+2ξ)×(βζ+2ω+2ρ+4ξ))−1,

and finally

(22) K4=β2γ2ρξ2ζ(C+D)((γ+2ξ)(γ+ω)(β+ρ)(β+2ρ)×(γ+ω+ξ)(γ+ω+2ξ)×(β+2ρ+4ξ)(β+2γ+2ρ)×(βζ+2ω+2ρ+2ξ)×(βζ+2ω+2ρ+4ξ))−1,

where *C* = *c*_0_ + *c*_1_ *β* + *c*_2_ *β* ^2^ + *c*_3_ *β* ^3^ is the same expression as in *K*_2_, and

(23) $$\begin{array}{c} D=2\xi \left(d_{0}+d_{1}\beta +d_{2}\beta^{2}\right), \end{array}$$

with

(24) d0=2(6ρ2+γ(3γ+4ω+6ρ)+ω(ω+8ρ+5ξ)+ξ(7γ+14ρ+4ξ)), d1=2(ρζ+3γ+4ω+5ρ+7ξ), d2=ζ+2.

Finally, it can be shown that *K*_2_ > *K*_1_, *K*_1_ > *K*_3_, *K*_2_ > *K*_4_, and *K*_4_ > *K*_3_.

### 3.1. Reduction of Transmission by Contact Investigation

Based on (19)–(22), we first numerically examine a collection of scenarios to determine the reduction in transmission due to contact investigation. Before examining the strategies of each individual and how these choices affect disease transmission (in the next subsection), we will assume that each individual discloses all of his or her contacts and thereby estimate the maximum disease reduction that can be achieved. We will examine low, moderate, and large within-group transmission, as measured by *μ* − 1, which is the expected number of secondary cases. Also, we will look at large and small values of the latency period relative to the infectious period, large and small values of the investigation rate *ξ* relative to the infectious period, large and small values of the protection fraction *ζ* for contacted susceptible individuals, and large and small values for the postexposure protection rate *ω*. These conditions are summarized in Table 2.

Table 2 suggests that the maximum benefit, in terms of prevented cases, occurs for intermediate transmission rates. Moreover, the table indicates that for this simple model, a long latent period, fast investigations, and prompt postexposure prophylaxis (unsurprisingly) favor disease control.

### 3.2. Effect of Nondisclosure on Disease Transmission

Each individual—Alice (the index case), Bob, or Charlie—may choose to disclose or not to disclose each of his or her two contacts. Thus, for example, Alice has four possibilities: (1) disclosing neither Bob nor Charlie, (2) disclosing Bob but not Charlie, (3) disclosing Charlie but not Bob, or (4) disclosing both Bob and Charlie. Each individual has four possible choices, and thus three individuals yield a total of 4^3^ = 64 possible choices.

How does the total number of transmitted cases *μ* − 1 change if some people fail to disclose contacts? We chose a scenario of rapid tracing and prophylaxis, together with a long latent period (Scenario 8 from Table 2, assuming an intermediate transmission rate *β* such that *μ*_0_ − 1 = 1). We computed *μ* − 1 for each of the 64 possible choice combinations, and these results are summarized in Table 3.

Is it, in general, possible for an individual to reduce his or her probability of disease by disclosure of others? By assumption, such a reduction is not possible for the first person infected in the group (Alice). Without loss of generality, we may consider the decrease in disease probability Charlie experiences if he (Charlie) discloses Alice, discloses Bob, or discloses both Alice and Bob. Since Alice and Bob each have four choices (disclosure or not of each of the other two people), a total of 16 possible combinations of these choices are available. For each specific choice of what Alice and Bob choose, we compare the infection probability when Charlie discloses Alice to the disease probability when Charlie discloses no one. The difference is the amount by which Charlie reduces his or her probability of disease by disclosing Alice compared to no one. Several salient facts are obtained from these expressions for contact investigations in a group of size 3.

First, in the three-person group, if Alice discloses no one, then Charlie can never reduce his likelihood of disease unless Bob discloses him. If neither Alice nor Bob is willing to disclose Charlie, then Charlie will never be known to the investigation before diagnosis. The only person Charlie should disclose to obtain benefit is Alice; since Alice is not disclosing Bob, Bob only discloses Charlie after he (Bob) is diagnosed and removed from transmission. But there is a possibility that Alice, who infected Bob, still has not been diagnosed yet; disclosure of Alice yields a possibility of benefit. The ability of Charlie, therefore, to benefit from disclosure depends on the choices made by the other persons in the network.

Equations (19) through (24) imply that Charlie in fact benefits (in terms of reduced disease) by disclosing Alice only when Charlie has been disclosed by Bob. Suppose that Bob does not disclose Charlie. Then the only opportunities for Charlie to disclose must occur if he is diagnosed or if Alice discloses him. Once Charlie is diagnosed, it is too late for him to benefit by disclosing someone else; if Alice discloses him, then Alice is already known to disease control investigators. If Alice is already known, there is no benefit to disclosing her again. Similarly, Charlie benefits by disclosing Bob only when Alice discloses Charlie. As before, the choices of the other group members affect not only the payoffs of Charlie, but also the ability of Charlie to benefit by making different choices.

Moreover, if *ζ* = 0, that is, susceptible people who have been contacted by an investigator are unable to become infected, Charlie does not benefit from disclosing anyone—one is able to obtain full protection without disclosing anyone once one is known to the investigation. In this simple model, effective prevention among people who have been investigated* reduces* incentives to disclose others, simply because there is nothing else for any given individual to gain once he or she has been investigated.

Finally, the expressions in the appendix show that (unsurprisingly), if there is no transmission (*β* = 0) or no investigation (*ξ* = 0), Charlie does not benefit from disclosure; no benefit from disclosure is seen when *γ* = 0 and *ρ* = 0, because the index case would never proceed to disease and diagnosis in these cases. Finally, Charlie never can increase his likelihood of disease by disclosure.

### 3.3. Tradeoffs between Disclosure and Disease

We now explore the model to determine the effect of costs of disease, disclosure, and participation. We first assume no overall participation costs or incentives (*C*_*o*_ = 0), so that the total cost for person *i* participating in an investigation consists only of the cost (real or perceived) that person *i* faces from disclosing each of the other persons who is disclosed. Alice (the initial case), unlike Bob and Charlie, cannot reduce her expected cost of disease by participating in an investigation.

#### 3.3.1. Benefits for Disclosure

Whenever the costs for disclosure are negative (there is a benefit to disclosure), the best strategy for each individual is to disclose all other individuals. Under these circumstances, disease prevention attains the maximum possible value. We assume a disease cost *F* of 1 (arbitrary units) and that *C*_*ij*_ = −*ϵF* (with *ϵ* > 1) and present numerical analysis for the scenario of long latency, prompt investigation, and prompt prophylaxis (Scenario 8, and assuming intermediate transmission rates as before, i.e. *μ*_0_ − 1 = 1). In this case, numerical analysis shows that the best strategy for each person is to disclose all contacts. Bob and Charlie each have an expected payoff of approximately 0.124 (arbitrary units) under these assumptions (i.e., Scenario 8, Table 2). If Bob switches to one of his other strategies, the expected payoff is lower: approximately −0.076 for disclosing neither, approximately 0.0243 for disclosing Charlie only, and approximately 0.0243 for disclosing Alice only. (The latter two payoffs in fact differ slightly, since Charlie may or may not be infected at the time of disclosure.) The same results are obtained for Charlie (Charlie receives a lower payoff if he changes to a different strategy), and, similarly, Alice receives a lower payoff if any other strategy other than disclosing everyone is chosen (in this case, simply because of the incentives for disclosure). Similar results were obtained for other values of *ϵ* (*ϵ* = 0.01, 0.002, and 0.001; results not shown).

#### 3.3.2. Costs for Disclosure

Where each individual may face costs for disclosing other individuals, the possibility of a conflict of interest arises. We will again assume the same numerical scenario as in the previous analysis (Scenario 8, intermediate transmission), except that we now add a small cost *ϵ* to disclosure. In this case, the Nash equilibrium is for each player to disclose no contacts, despite the fact that this yields the largest possible transmission of disease. This occurs because the index case Alice, already infected, can never reduce her probability of infection by disclosing and so has no incentive to bear the cost of disclosure. The personal cost is minimized by never disclosing. Unfortunately, Bob will never be disclosed by Alice and will in turn only be contacted after diagnosis—after which time it is also too late to benefit directly by disclosure. The costs are also minimized for Bob by never disclosing. The analysis is the same for Charlie. Thus, the other individuals will never be contacted before infection, and the same logic will apply to them. No contact tracing will occur, and preventable infections will happen.

When we assume a reward for disclosing at least one contact (17), a different result may occur. This is an assumption that an individual has an incentive to disclose at least one person, but no more. Assuming, for example, *C*_*o*_ = −0.15 and *C*_*ij*_ = 0.1 for all *i*, *j*, *i* ≠ *j*, we find two solutions (in the sense that changing strategies cannot yield an improvement), as shown in Table 4.

For the first strategy in Table 4, we find that Alice should disclose Bob, Bob should disclose Charlie, and Charlie should disclose Alice. By definition, this is equilibrium, because no one can benefit by departing from it provided the other participants do not change strategies. For Alice, the only difference in payoff results from costs and incentives related to disclosure; Alice cannot affect her own infection status (as the index case, by assumption). By assumption, Alice receives a benefit for disclosing one person but experiences a net cost for disclosing two. Alice could choose either to disclose.

Bob and Charlie have the same incentives that Alice has to disclose exactly one other person. For Bob and Charlie, however, disclosing others may affect the probability of disease, and so the best depends not only on the costs or incentives for disclosure, but also on disease transmission. If the strategy of Alice is to disclose Bob, then the best strategy for Bob is to disclose Charlie and not Alice. Alice is the index case and willing to disclose Bob, and so it would frequently be wasteful for Bob to disclose Alice—Alice is likely to have already been diagnosed. Similarly, if Alice is choosing to disclose Bob, Charlie benefits more from disclosing Alice than Bob. If Bob (but not Alice) is using the strategy of disclosing Charlie, then Charlie could infer that whenever he has been investigated before infection, it was the result of Bob's disclosure and that disclosing Bob again is counterproductive—Alice is the better choice.

### 3.4. Disclosure before Diagnosis

In the preceding section, individuals are assumed to use the same strategy for disclosure all the time, whether or not the person was identified before he or she became a case, or after. In the latter case, the individual has no chance to prevent her or himself from becoming diseased. We next suppose that each individual could make a different choice about disclosure depending on whether or not the person was originally identified as a result of seeking health care (diagnosed from state *I*), or as a result of investigation. We keep the same model of transmission, but now distinguish between removed cases. We denote by *R* cases diagnosed from the state *I*, and we denotr by *R*′ cases known prior to symptoms (individuals in state *E*′ who become diseased). The revised equations are given in Appendix. Here, *X*_1_, *X*_2_, and *X*_3_ range through the set *S*, *E*, *I*, *R*, *S*′, *E*′, *R*′, and *V*. The variable *δ*_*ij*_ is 1 if person *i* chooses to disclose person *j* if person *i* is identified before symptoms and 0 otherwise, and *δ*_*ij*_′ is 1 if person *i* chooses to disclose person *j* after person *i* has symptoms, and 0 if person *i* does not disclose person *j* under these circumstances. If *δ*_*ij*_ = *δ*_*ij*_′ for all *i*, *j*, this model reduces to the model previously analyzed. For *N* = 3 people, each person has 2 choices for each contact, for four decision variables. With three modeled individuals, there are thus 2^12^ = 4096 strategic choice combinations, and we simply confine our attention to a few special cases of interest. The Kolmogorov equations will be numerically integrated using the  lsoda function in the R package  deSolve, v. 1.10. We assume no participation incentives in this section (*C*_*o*_ = 0).

If no one discloses after diagnosis (*δ*_12_′ = *δ*_21_′ = *δ*_13_′ = *δ*_31_′ = *δ*_23_′ = *δ*_32_′ = 0), then no investigations ever result and the values of *δ*_*ij*_ do not matter. On the other hand, if everyone always discloses after diagnosis (*δ*_12_′ = *δ*_21_′ = *δ*_13_′ = *δ*_31_′ = *δ*_23_′ = *δ*_32_′ = 1), then the first person to become diagnosed discloses both other individuals. There is no further benefit to disclosing these people again. Numerically, we chose Scenario 8 from Table 2 and assigned all disclosure costs to equal 0.1 times the cost of disease. The sole Nash equilibrium found is for neither Bob nor Charlie to disclose anyone else if contacted. Similar results were obtained even when the cost was as low as 0.0001 for disclosure (the lowest positive cost we numerically examined).

We also examined the case *δ*_12_′ = 1 and = *δ*_21_′ = *δ*_13_′ = *δ*_31_′ = *δ*_23_′ = *δ*_32_′ = 0, where the index case Alice will initiate the investigation by disclosing Bob if diagnosed. Assuming no incentives for participation, it is optimal for Bob to disclose Charlie provided that the disclosure cost is very small. We found, using Scenario 8 from above, that for a tiny cost of 10^−4^, Bob should disclose Charlie and Charlie should disclose no one. Bob should disclose Charlie because Charlie may be diagnosed sooner than otherwise, reducing the risk to Bob. Charlie should not disclose anyone; the only way Charlie could ever be investigated is either if he is diagnosed or if he is disclosed by Bob. If Charlie has been diagnosed already, it is too late for him to act to prevent disease and so disclosure no longer is beneficial for him; if Bob has disclosed Charlie already, there is no benefit to disclosing Bob again; either way, Charlie should not disclose Bob in this case. Moreover, Bob only discloses Charlie when Bob has been contacted prior to disease, and this only occurs when Alice has already been diagnosed. Thus, Charlie will only be contacted when both Alice and Bob have already been investigated, and the solution to the equations confirms the optimal strategy for Charlie. This result, however, disappears when the cost of disclosure is raised. At a cost of 10^−3^, the optimal strategy is for Bob to disclose no one; Charlie will never be faced with the choice of what to do if investigated before diagnosis.

## 4. Discussion

In this paper, we analyzed a simple model of cost-benefit tradeoffs in a stylized model of contact investigation and disclosure, reflecting public health circumstances in which individuals may not wish to disclose other individuals in their contact network. Such circumstances may arise if such contacts reflect illicit activity, undocumented presence in the country, or other reasons related to privacy. We therefore assumed a cost for each such disclosure. The model assumes that individuals may benefit by disclosing other individuals in their network of contacts and that the sole such benefit is a reduction in infection risk resulting from earlier diagnosis of other individuals in the group. We assumed a specific simple form where individuals use a fixed strategy, for which any specific contact may or may not be disclosed, and that this did not depend on the progression of the epidemic. We assumed a simple stochastic epidemic model where individuals could be protected after exposure by vaccination, and that once an individual is diagnosed, he or she is removed and will transmit no more infection. Finally, our analysis was restricted to the case of a simple three person cluster. We developed the analytic Kolmogorov equations for the stochastic process, and solved these equations to determine the expected payoffs.

In this setting, we found that if all individuals have a cost of disclosure with no participation incentives, then the optimal individual decision is to simply not disclose others. Contact investigation is unsuccessful, and more transmission within the group results. Also, the population *R*_0_ is larger than it would otherwise have been. In this case, optimal personal decisions are suboptimal for the group as a whole. In this simple model, our assumptions guaranteed that once an individual is infected, then the only remaining component in their game payout that can induce disclosure is his or her fixed cost or benefit of disclosure. Thus, in our base case analysis, the index case has no incentive to disclose—it is too late for him or her to benefit. No one else is disclosed, and thus each other individual likewise is only diagnosed after infection, also too late to benefit.

However, if there is some benefit to disclosing—some incentive to remove all or part of these costs—a different structure emerges. We examined additional cases: (1) completely offsetting the costs of disclosure and (2) partially offsetting the costs of disclosure. If the costs of disclosure are completely offset, so that all individuals benefit from disclosing, the unsurprising result was that all individuals disclose all contacts; this results in a smaller *R*_0_ and an alignment of individual and group optimality.

We also examined a case of partially offset costs, in which a person should disclose one such contact, but not both. In this case, we found two solutions. Using the conventional names Alice (for the index case), Bob, and Charlie, these are as follows. If Alice disclosed Bob, then Bob should disclose Charlie, and Charlie should disclose Alice. Similarly, if Alice disclosed Charlie, then Charlie should disclose Bob, and Bob should disclose Alice; the same pattern is seen, with the roles of Bob and Charlie reversed.

We examined an extended version of the model in which individuals could make a different choice depending on whether they were identified in time to prevent illness. This model found that the direct benefits of prevention could outweigh small disclosure costs, favoring disclosure. While this threshold for favoring disclosure may be larger for alternative or more realistic model structures, we believe that direct immediate prevention benefits should not be relied on to provide sufficient incentives for participation. Reducing costs—including perceived costs—is crucial.

In real outbreaks, individuals lack the information necessary to weigh the risks and benefits of disclosure. Individuals do not, in general, know the extent of their exposure, the benefits of vaccination at different times, nor the benefit they would receive by disclosure. Thus, the solutions to the game model are idealized optimal strategies realizable under perfect information. Importantly, this analysis, focusing as it does only on the small group (of size 3) and not beyond, does not fully reflect the epidemiology of novel pathogen introduction. Here, failure to prevent transmission early may lead to widespread transmission beyond the small group. The analysis presented above only includes transmission within the small contact group and could be straightforwardly extended to take into account the benefits—epidemiological and otherwise—of stopping a large epidemic. We also note that real decision making could take into account a much richer strategic set, so that individuals could have a different strategy depending on whether or not they know how many cases there have been, what other individuals have done, or other factors (e.g. [46, 61, 63, 70–73]). Finally, the limitations of modeling human behavior as governed by classical economic models of rational optimization have long been noted [74]; in disease control settings or emergency responses more generally, fear [75] as well as altruism [73, 76, 77] have been reported.

Our model does show how decision-making based solely on reducing an individual's direct risk of disease can lead to noncompliance and an overall unfavorable outcome for the group. Moreover, it suggests that the ability of an individual to reduce his or her own risk would, under these assumptions, be expected to reduce compliance with contact investigation. The findings highlight the central importance of reducing costs of contact investigation for all participants, perhaps through incentives. Further work will be needed to assess the robustness of these conclusions. Empirical data on perceptions of the risks and benefits of contact investigation and the reasons for compliance and noncompliance are urgently needed.

## Figures and Tables

**Table 1:** Reduction in infection for Charlie due to disclosures by Charlie, assuming given strategies for the other two individuals. For each row, Alice is assumed to disclose either Bob or Charlie or both, as indicated in the first two columns; Bob is assumed to disclose either Alice or Charlie or both, as given in the next two columns. The next column (Alice versus none) shows how much the infection probability for Charlie is reduced by disclosing Alice instead of disclosing no one. The column labeled “Bob versus none” shows the reduction by disclosing Bob instead of no one and so forth. Analytic expressions for the quantities *K*_1_, *K*_2_, *K*_3_, and *K*_4_ are given in the text.

Alice discloses		Bob discloses		Reduction in infection probability for Charlie comparing disclosure choices of				
Bob	Charlie	Alice	Charlie	Alice versus none	Bob versus none	Both versus none	Both versus Alice	Both versus Bob
N	N	N	N	0	0	0	0	0
Y	N	N	N	0	0	0	0	0
N	Y	N	N	0	*K*_2_	*K*_2_	*K*_2_	0
Y	Y	N	N	0	*K*_4_	*K*_4_	*K*_4_	0
N	N	Y	N	0	0	0	0	0
Y	N	Y	N	0	0	0	0	0
N	Y	Y	N	0	*K*_2_	*K*_2_	*K*_2_	0
Y	Y	Y	N	0	*K*_4_	*K*_4_	*K*_4_	0
N	N	N	Y	*K*_1_	0	*K*_1_	0	*K*_1_
Y	N	N	Y	*K*_1_	0	*K*_1_	0	*K*_1_
N	Y	N	Y	*K*_1_	*K*_2_	*K*_2_ + *K*_1_	*K*_2_	*K*_1_
Y	Y	N	Y	*K*_1_	*K*_4_	*K*_4_ + *K*_1_	*K*_4_	*K*_1_
N	N	Y	Y	*K*_3_	0	*K*_3_	0	*K*_3_
Y	N	Y	Y	*K*_3_	0	*K*_3_	0	*K*_3_
N	Y	Y	Y	*K*_3_	*K*_2_	*K*_2_ + *K*_3_	*K*_2_	*K*_3_
Y	Y	Y	Y	*K*_3_	*K*_4_	*K*_4_ + *K*_3_	*K*_4_	*K*_3_

**Table 2:** Numerical scenarios showing the percent reduction in disease transmission achievable by contact investigation and postexposure prophylaxis, assuming complete disclosure. The latency column provides the ratio of the expected duration of the latent period relative to the infectious period, the tracing column is the ratio of the duration of the infectious period to the expected waiting time to be found from a single disclosure, and the prophylaxis column is the ratio of the duration of the latent period to the waiting time to postexposure prophylaxis following contact investigation. The percent decline in transmission within the group ((1 − ((μ_1_ − 1)/(μ_0_ − 1))) × 100%) is shown for low, medium, and high within-group transmission (μ_0_ − 1 = 0.1, 1, and 1.9). No protective effect against infection was assumed for susceptible individuals who were interviewed by contact investigators (ζ = 1).

Scenario	Latency	Tracing	Prophylaxis	Reduction (%)
	ρ/γ	ξ/ρ	ω/γ	μ − 1 = 0.1,1, 1.9
1	0.1	0.1	0.1	0.543%, 1.15%, 0.0832%
2	10	0.1	0.1	6.89%, 12.7%, 7.01%
3	0.1	10	0.1	4.36%, 13.7%, 3.43%
4	10	10	0.1	12%, 21.6%, 11.9%
5	0.1	0.1	10	0.791%, 1.76%, 0.267%
6	10	0.1	10	47.8%, 53.1%, 49.8%
7	0.1	10	10	8.64%, 19%, 9.71%
8	10	10	10	82.8%, 84.9%, 83.1%

**Table 3:** Expected number of secondary cases for different disclosure choices (Scenario 8, Table 2, with μ_0_ − 1 = 1). The rows indicate the persons disclosed by Alice and Charlie; the columns indicate who Bob discloses. The cell entries indicate the expected number of secondary cases within the group, obtained by integrating (6).

	Charlie discloses	Bob discloses	Bob discloses	Bob discloses	Bob discloses
		Neither	Alice only	Charlie only	Both
Alice discloses neither	Neither	1	0.998	0.835	0.835
	Alice only	0.998	0.997	0.834	0.834
	Bob only	0.835	0.834	0.671	0.671
	Both	0.835	0.834	0.671	0.671

Alice discloses Bob only	Neither	0.508	0.506	0.158	0.158
	Alice only	0.506	0.505	0.157	0.157
	Bob only	0.506	0.505	0.157	0.157
	Both	0.506	0.505	0.157	0.156

Alice discloses Charlie only	Neither	0.508	0.506	0.506	0.506
	Alice only	0.506	0.505	0.505	0.505
	Bob only	0.158	0.157	0.157	0.157
	Both	0.158	0.157	0.157	0.156

Alice discloses both	Neither	0.156	0.155	0.154	0.154
	Alice only	0.155	0.153	0.152	0.152
	Bob only	0.154	0.152	0.152	0.151
	Both	0.154	0.152	0.151	0.151

**Table 4:** Nash equilibrium strategies resulting when *C*_*o*_ = −0.15 and *C*_*ij*_ = 0.1.

	Alice	Bob	Charlie	Total transmission (μ − 1)
	Discloses	Discloses	Discloses	
1	Bob only	Charlie only	Alice only	0.157
2	Charlie only	Alice only	Bob only	0.157

## Appendix

The extended equations for the analysis in Section 3.4 are as follows:

(A.1) ddtpX1,X2,X3 =γ(1X1=I−1X1=E)pE,X2,X3+γ(1X2=I−1X2=E)pX1,E,X3  +γ(1X3=I−1X3=E)pX1,X2,E  +ρ(1X1=R−1X1=I)pI,X2,X3  +ρ(1X2=R−1X2=I)pX1,I,X3  +ρ(1X3=R−1X3=I)pX1,X2,I  +λ(X1,X2,X3)(1X1=E−1X1=S)pS,X2,X3  +λ(X1,X2,X3)(1X2=E−1X2=S)pX1,S,X3  +λ(X1,X2,X3)(1X3=E−1X3=S)pX1,X2,S  +γ(1X1=R′−1X1=E′)pE′,X2,X3  +γ(1X2=R′−1X2=E′)pX1,E′,X3  +γ(1X3=R′−1X3=E′)pX1,X2,E′  +ω(1X1=V−1X1=S′)pS′,X2,X3  +ω(1X2=V−1X2=S′)pX1,S′,X3  +ω(1X3=V−1X3=S′)pX1,X2,S′  +ω(1X1=V−1X1=E′)pE′,X2,X3  +ω(1X2=V−1X2=E′)pX1,E′,X3  +ω(1X3=V−1X3=E′)pX1,X2,E′  +ζλ(X1,X2,X3)(1X1=E′−1X1=S′)pS′,X2,X3  +ζλ(X1,X2,X3)(1X2=E′−1X2=S′)pX1,S′,X3  +ζλ(X1,X2,X3)(1X3=E′−1X3=S′)pX1,X2,S′  +ξ(δ211X2∈{S′,E′,R′,V}+δ311X3∈{S′,E′,R′V})  ×((1X1=S′−1X1=S)pS,X2,X3+(1X1=E′−1X1=E)pE,X2,X3    +(1X1=R−1X1=I)pI,X2,X3)  +ξ(δ121X1∈{S′,E′,R′,V}+δ321X3∈{S′,E′,R′,V})  ×((1X2=S′−1X2=S)pX1,S,X3+(1X2=E′−1X2=E)pX1,E,X3    +(1X2=R−1X2=I)pX1,I,X3)  +ξ(δ131X1∈{S′,E′,R′,V}+δ231X2∈{S′,E′,R′,V})  ×((1X3=S′−1X3=S)pX1,X2,S+(1X3=E′−1X3=E)pX1,X2,E    +(1X3=R−1X3=I)pX1,X2,I)  +ξ(δ21′1X2=R+δ31′1X3∈R)  ×((1X1=S′−1X1=S)pS,X2,X3+(1X1=E′−1X1=E)pE,X2,X3    +(1X1=R−1X1=I)pI,X2,X3)  +ξ(δ12′1X1=R+δ32′1X3=R)  ×((1X2=S′−1X2=S)pX1,S,X3+(1X2=E′−1X2=E)pX1,E,X3    +(1X2=R−1X2=I)pX1,I,X3)  +ξ(δ13′1X1=R+δ23′1X2=R)  ×((1X3=S′−1X3=S)pX1,X2,S+(1X3=E′−1X3=E)pX1,X2,E    +(1X3=R−1X3=I)pX1,X2,I).
